# Overexpression of SIRT1 in Rat Skeletal Muscle Does Not Alter Glucose Induced Insulin Resistance

**DOI:** 10.1371/journal.pone.0121959

**Published:** 2015-03-23

**Authors:** Amanda E. Brandon, Jennifer Tid-Ang, Lauren E. Wright, Ella Stuart, Eurwin Suryana, Nicholas Bentley, Nigel Turner, Gregory J. Cooney, Neil B. Ruderman, Edward W. Kraegen

**Affiliations:** 1 Diabetes and Metabolism Division, Garvan Institute of Medical Research, 384 Victoria St., Darlinghurst, NSW, 2010, Australia; 2 UNSW Medicine, University of New South Wales, Sydney, Australia; 3 Boston University School of Medicine, Boston, Massachusetts, United States of America; Tohoku University, JAPAN

## Abstract

SIRT1 is a NAD^+^-dependent deacetylase thought to regulate cellular metabolic pathways in response to alterations in nutrient flux. In the current study we investigated whether acute changes in SIRT1 expression affect markers of muscle mitochondrial content and also determined whether SIRT1 influenced muscle insulin resistance induced by acute glucose oversupply. In male Wistar rats either SIRT1 or a deacetylase inactive mutant form (H363Y) was electroprated into the tibialis cranialis (TC) muscle. The other leg was electroporated with an empty control vector. One week later, glucose was infused and hyperglycaemia was maintained at ~11mM. After 5 hours, 11mM glucose induced significant insulin resistance in skeletal muscle. Interestingly, overexpression of either SIRT1 or SIRT1 (H363Y) for 1 week did not change markers of mitochondrial content or function. SIRT1 or SIRT1 (H363Y) overexpression had no effect on the reduction in glucose uptake and glycogen synthesis in muscle in response to hyperglycemia. Therefore we conclude that acute increases in SIRT1 protein have little impact on mitochondrial content and that overexpressing SIRT1 does not prevent the development of insulin resistance during hyperglycaemia.

## Introduction

Sirtuin 1 (SIRT1) is a NAD^+^-dependent deacetylase with a large range of target proteins that are important for apoptosis, the cell cycle, circadian rhythms, mitochondrial function, and metabolism [1]. SIRT1 is thought to be nutritionally regulated and be responsible for the beneficial effects of calorie restriction [2,3]. Levels of SIRT1 are reportedly decreased with high-fat feeding and may therefore have a role in lipid-induced insulin resistance [4]. Consistent with these results, *in vitro* studies has shown that SIRT1 is down regulated under hyperglycaemic conditions in liver [5,6], endothelial [7,8], mesangial [9], corneal epithelial [10] and C2C12 muscle cells [11]. Rescuing the decrease in SIRT1 via pharmacological intervention (e.g. resveratrol) or protein overexpression can reverse the detrimental hyperglycaemic effect in these systems [8–10].

Using a model of hyperglycaemia (~11mM blood glucose) generated by a moderate intravenous glucose infusion into rats, we have shown previously that skeletal muscle insulin resistance consistently develops between 3 and 5h [12–14]. Interestingly, this insulin resistance developed prior to alterations in the insulin signaling pathway [12,13] but occurred in association with increased glycogen content and reduced AMPK activity [12,14]. In an attempt to further delineate the underlying mechanism(s), Saha *et al* [15] incubated EDL muscle strips in 5 or 25 mM glucose and found that the lactate to pyruvate ratio was increased, indicative of a decrease in the NAD^+^/NADH ratio. A tendency for SIRT1 protein levels to de decreased (20%, p = 0.10) was also found after incubation in this study [15]. Thus, combining the results from cell work and the muscle strip experiments, it is possible that SIRT1 may contribute to hyperglycemia-induced insulin resistance. Hence, the aim of the current study was to investigate whether SIRT1 overexpression would prevent the development of insulin resistance in skeletal muscle *in vivo*.

## Methods

### Cell culture

All cell culture reagents are from Life Technologies (Auckland, NZ) unless otherwise stated. Mouse skeletal muscle cell line C2C12 myoblasts were maintained in DMEM/F21 (1:1) with 10% FBS, 100 U/ml penicillin and 100 U/ml streptomycin (growth medium) at 37°C under a 5% CO_2_ atmosphere. For transfection, the cells were grown on 6-well plates (Corning Inc., NY, USA) in 2 ml of growth medium. Once at 90% confluence, cells were transfected with either empty plasmid (2μg), a truncated but fully active form of SIRT1 [16], or a version of the SIRT1 construct that has a point mutation that renders it deacetylase inactive (SIRT1 (H363Y); kind gifts from Aimin Xu [16]) using the X-tremeGENE HP DNA transfection reagent (Roche, Mannheim, Germany) and opti-MEM reagent. Cells were collected 24h post transfection. Data presented are an average of 3 independent experiments.

### Animals

All surgical and experimental procedures performed were approved by the Garvan Institute/St Vincent’s Hospital Animal Ethics Committee and were in accordance with the National Health and Medical Research Council of Australia’s guidelines on animal experimentation.

Adult male Wistar rats (Animal Resources Centre, Perth, Australia) were communally housed in temperature controlled (22 ± 0.5°C) 12 h light-dark cycle rooms. Rats were fed *ad libitum* a standard chow diet (Rat Maintenance Diet; Gordon Specialty Feeds, Sydney, Australia). Rats were acclimatized for 1 week prior to surgery.

#### *In Vivo* Electroporation (IVE) and Surgical Procedures

After the acclimatization period, rats were electroporated as previously described [17–19]. Briefly, under anaesthesia, control and test muscles were pretreated for 2 h with 90 units of hyaluronidase to break down components of the extracellular matrix to improve transfection efficiency [20]. Either SIRT1, or SIRT1 (H363Y), was injected into the test (right) tibialis cranialis (TC), and empty plasmid was injected into the control (left) TC via 6 x 50ul injections. Both legs underwent an electroporation protocol consisting of one 800 V/cm, 100 ms pulse followed by four 80 V/cm, 100 ms pulses at 1 Hz. Immediately after the IVE, while still under anaesthesia, dual cannulation of both jugular veins was performed as described previously [12,21].

#### Glucose Infusion

Seven days after surgery, rats (approximately 300g body weight) were randomly divided into treatment groups. After a basal blood sample was taken, a 50% (w/v) glucose infusion commenced. Rats were infused for either 0 or 5h using a peristaltic roller pump (101U/R; Watson-Marlow, Falmouth, UK). Blood samples were taken every 30 min and the glucose infusion rate was altered to maintain blood glucose concentration at ~11 mM. Red blood cells from each sample were resuspended in heparinised saline and returned to the animal. 2-deoxy-d-[2,6-^3^H]glucose and [U-^14^C]glucose (PerkinElmer, Melbourne, Australia) were administered as an intravenous bolus in the last 30 min of the glucose infusion. Blood samples were taken 2, 5, 10, 15, 20 and 30 min after administration of the tracer bolus for estimation of tracer clearance and blood glucose. Animals were then euthanized and tissues were rapidly removed, freeze-clamped, and stored at -80°C for later analysis. TC muscle was powdered prior to any assay procedure to ensure homogeneity.

### Analytic Methods

Blood and plasma glucose levels (YSI2300; Yellow Springs Instruments, Yellow Springs, OH, USA), and plasma insulin (Rat RIA, Millipore, Missouri, USA) were measured. Plasma and tissue levels of ^3^H- and ^14^C-labelled tracers were measured to calculate whole body glucose disposal rate (*R*_d_), to estimate tissue glucose uptake (*R*_g_′), and to measure glucose incorporation rate into glycogen. Assays and calculations for the glucose disappearance, glucose uptake into tissues and glucose incorporation into glycogen and glycogen content measures are as previously described [22]. Oxidation of palmitate and glutamate was assessed in muscle tissue homogenates as described previously [23]. Enzyme activities for citrate synthase (CS), β-hydroxyacyl CoA dehydrogenase (βHAD) and succinate dehydrogenase (SDH) were done as previously described [23,24]. Mitochondrial measurements were performed on animals in the basal state (without glucose infusion).

### Immunoblotting

#### Protein Extraction

24h post transfection, cells were washed once with PBS and collected in RIPA buffer (65 mM Tris (pH 7.4), 150 mM, NaCl, 5 mM EDTA, 1% Nonidet P-40, 0.5% sodium deoxycholate, 0.1% SDS, 10% glycerol, 1 μg/ml aprotinin, 1 μg/ml leupeptin, 10mM sodium fluoride, 1 mM Na_3_VO_4_, 1 mM PMSF and 50mM nicotinamide) and snap frozen. On the first thaw, cells were sonicated and incubated for 1h. For the TC muscles approximately 50 mg of powdered TC was homogenised in RIPA buffer and incubated for 1–2h. Lysates from cells and tissues were centrifuged at 12,000 g to remove any insoluble particles and protein concentration was determined via a protein assay (BioRad, Hercules, CA, USA).

#### Immunoblot analysis

Cell and tissue lysates were subjected to SDS-PAGE, transferred to PVDF membranes, blocked in 2–5% BSA and then immunoblotted with antibodies for SIRT1, Acetyl-p53 (K379), p53, insulin receptor, p-Akt (S473), Akt, pACC (S79), ACC, VDAC/Porin (all from Cell Signaling), p-insulin receptor (Y1162/3; Invitrogen), OXPHOS (complexes I, II, III, V; MitoSciences), complex IV (Molecular Probes). Densitometry analysis was performed using ImageJ software (NIH; http://imagej.nih.gov/ij/).

### Statistics

Data are expressed as means ± SEM. Differences between groups were determined by paired students t-test, one-, or two-way ANOVA as appropriate (see figure legends). If the one-way ANOVA reached significance a Newman-Keuls multiple comparison post hoc test was conducted. If the two-way ANOVA reached significance a Bonferroni's post hoc test was conducted. All statistical analysis was performed using GraphPad Prism (Version 6 for Windows, GraphPad Software, San Diego, California, USA). The level of significance was p≤0.05.

## Results

### In Vitro

**Fig 1:**
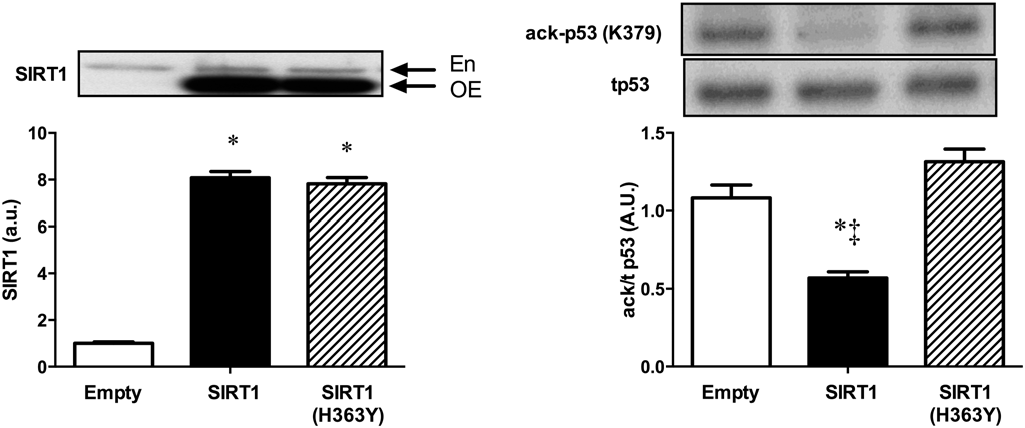
Effect of SIRT1 or SIRT1 (H363Y) overexpression on acetylation of p53 in C2C12 myoblasts. (A) Protein expression of SIRT1 or SIRT1 (H363Y) in C2C12 myoblasts; (B) acetyl-p53 (K376) in C2C12 myoblasts overexpressing empty vector, SIRT1 or SIRT1 (H363Y). *p<0.01 vs empty vector; ‡ p<0.01 vs SIRT1 (H363Y), one-way ANOVA. En, endogenous SIRT1 protein; OE, overexpressed SIRT1 protein. Data are expressed as means ± SEM. n = 3 replicate experiments done in triplicate.

To confirm the activity of the constructs, C2C12 myoblasts were successfully transfected with SIRT1, a deacetylase inactive mutant of SIRT1 (H363Y) or empty vector for 24h (Fig. 1A). The tumour suppressor protein p53 is a well-described target of SIRT1 [1]. There was a decrease in acetyl-p53 in the SIRT1 overexpressing myoblasts when compared to the empty vector and no change with the mutated version (Fig. 1B). Thus we confirmed the activity of the constructs and subsequently used them to increase SIRT1 protein in skeletal muscle of rats.

### In Vivo

#### Basal

**Fig 2:**
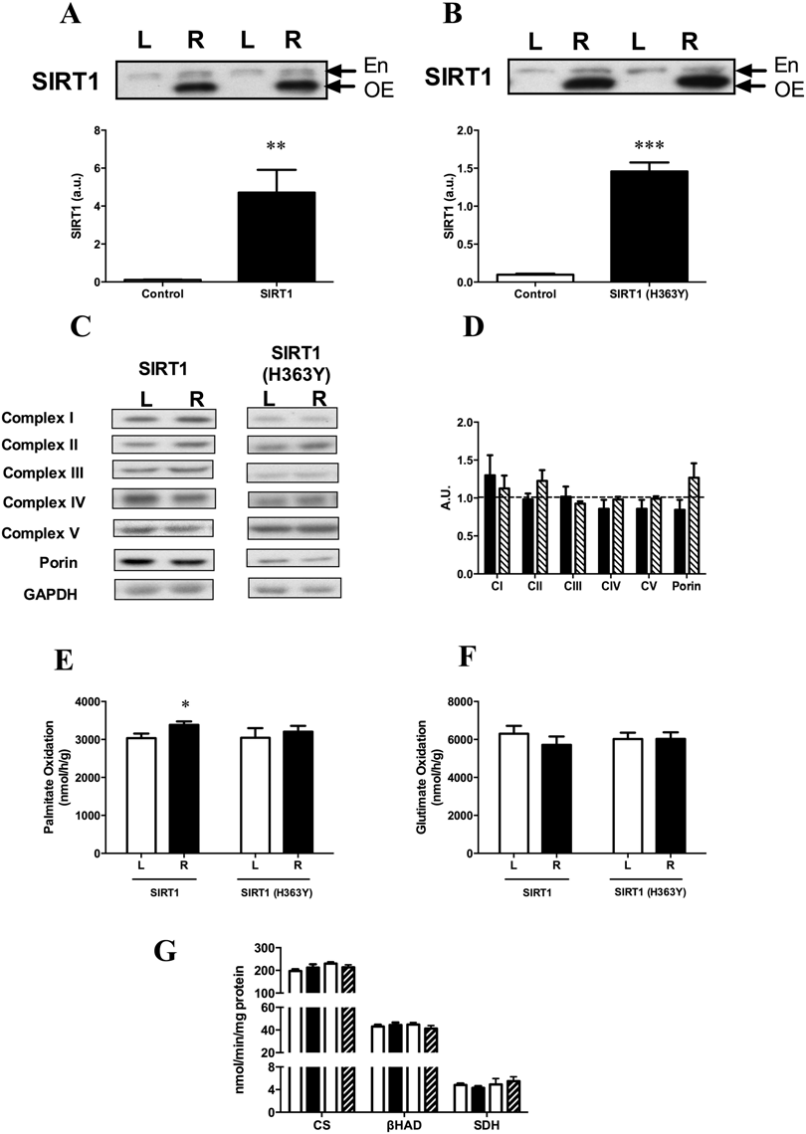
Effect of SIRT1 and SIRT1 (H363Y) overexpression on mitochondrial proteins and function in the basal state. (A) SIRT1 and (B) SIRT1 (H363Y) over expression in TC muscle. (C) Representative immunoblots for mitochondrial proteins and (D) densotrometric quantitation of these protein blots expressed as relative to the control leg (dotted line; Black bars SIRT1; Hatched bars SIRT1 (H363Y)). (E) Oxidation rates of muscle homogenates incubated in medium containing palmitate or (F) glutamate. (G) Enzyme activities for citrate synthase (CS), β-hydroxyacyl CoA dehydrogenase (βHAD) and succinate dehydrogenase (SDH). * p = 0.049; **p<0.01; ***p<0.001 vs control leg, paired t-test. En, endogenous SIRT1 protein; OE, overexpressed SIRT1 protein. Data are expressed as means ± SEM. n = 5–12 animals.

Electroporation of the SIRT1 and SIRT1 (H363Y) constructs significantly increased the levels of these proteins in the test leg (right) compared to the control leg (left; Fig. 2A, 2B). To determine if overexpression of either of the SIRT1 constructs altered mitochondrial parameters we examined the content of the mitochondrial respiratory chain and porin, an abundant mitochondrial protein, often used as a marker of mitochondrial density [25,26] in control muscle and muscle overexpressing the SIRT1 constructs. There was no difference in these parameters in the left and right tibialis muscles (Fig. 2C and D). As a measure of function, whole tissue homogenate oxidation rates of palmitate and glutamate were measured. Palmitate oxidation in tissue homogenates showed a small 11% increase in the SIRT1 overexpressing leg that was statistically significant using a paired t-test (p = 0.049). There was no change in the SIRT1 (H363Y) overexpressing leg (Fig. 2E). When glutamate was used no difference in substrate oxidation was observed in either the SIRT1 or SIRT1 (H363Y) overexpressing leg (Fig. 2F). Enzyme activities for CS, βHAD and SDH were all not different from the control leg for either SIRT1 or the deacetylase mutant form (Fig. 2G). This data suggests mitochondrial content and function were largely unaltered by the overexpression of either an active or inactive version of SIRT1 for 1 week.

#### Glucose Infusion

**Fig 3:**
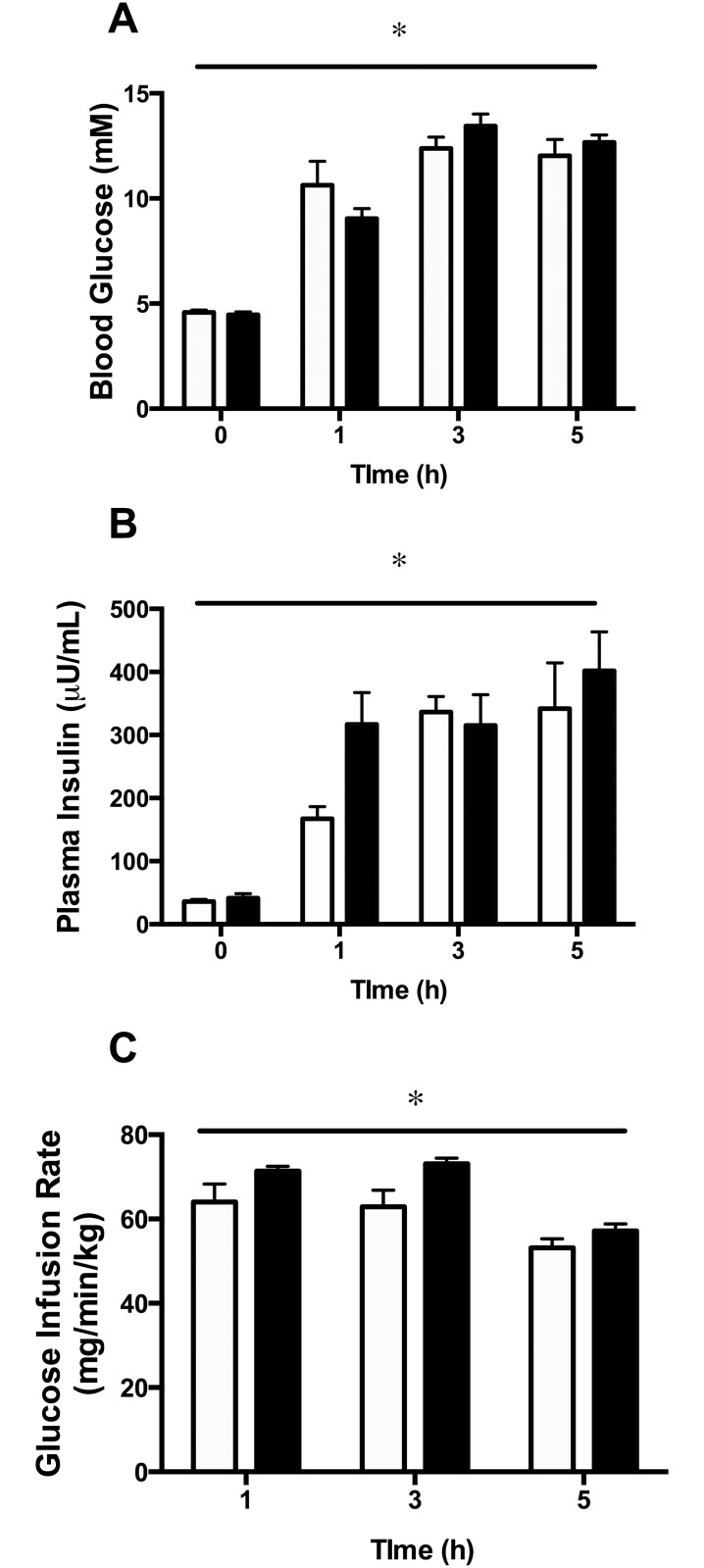
Blood glucose (A), plasma insulin (B) and glucose infusion rate (C) in animals overexpressing SIRT1 (white bars) or SIRT1 H363Y (black bars) in the right TC muscle. *p<0.001 for the main effect of glucose infusion using a two-way repeated measures ANOVA. There was no significant interaction between glucose infusion and construct for any parameter measured. Data are expressed as means ± SEM. n = 4–10 animals.

Blood glucose levels increased significantly in glucose-infused animals and remained stable over the 5h infusion period (Fig. 3A). Plasma insulin was also elevated and remained stable during glucose infusion (Fig. 3B). The amount of glucose infused to maintain hyperglycemia decreased between 3h and 5h of infusion, indicating the existence of whole-body insulin resistance at 5h (Fig. 3C).

**Fig 4:**
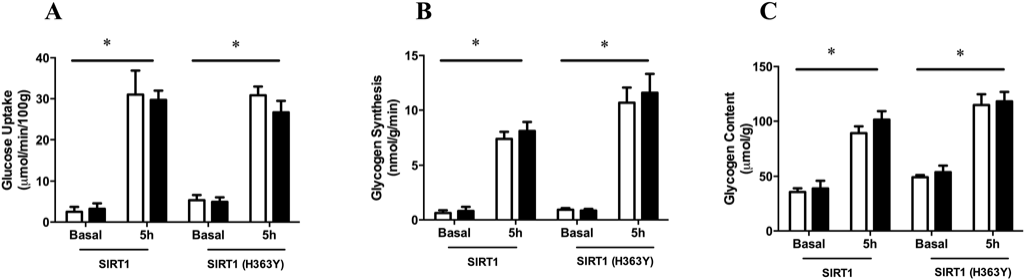
Effect of SIRT1 and SIRT1 (H363Y) overexpression on skeletal muscle insulin sensitivity. (A) Glucose uptake, (B) glycogen synthesis and (c) glycogen content of control (white bars) or overexpressing (Black bars) muscle. *p<0.01 for the main effect of glucose infusion using a two-way ANOVA. There was no significant interaction between glucose infusion and construct for any parameter measured. Data are expressed as means ± SEM. n = 4–12 animals.

After 5h glucose infusion, tibialis muscle glucose uptake was assessed as previously described [12–14]. Overexpression of SIRT1 or SIRT1 (H363Y) for 7 days had no effect on glucose uptake into tibialis muscle (Fig. 4A). There were also no differences between the test and control legs in glycogen synthesis or content after 5h infusion, irrespective of which construct was overexpressed (Fig. 4B and C).

**Fig 5:**
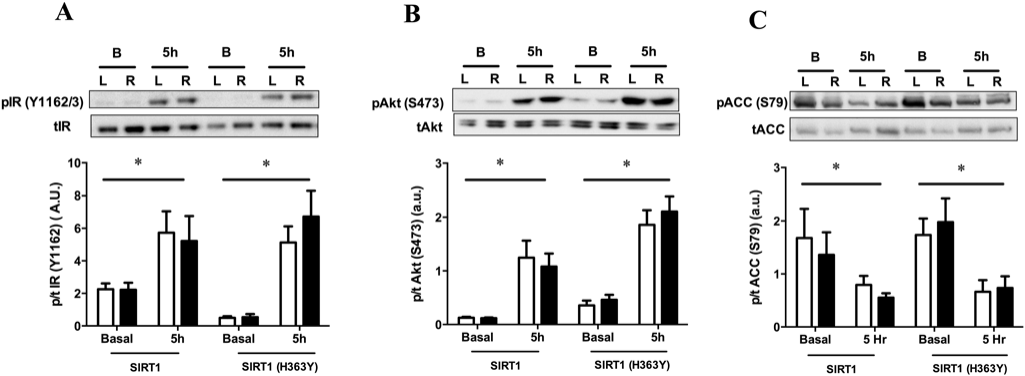
Effect of SIRT1 and SIRT1 (H363Y) overexpression on skeletal muscle signaling intermediates. (A) Insulin receptor (IR), (B) Akt and (C) acetyl-CoA carboxylase (ACC). *p<0.05 for the main effect of glucose infusion using a two-way ANOVA. There was no significant interaction between glucose infusion and construct for any parameter measured. Data are expressed as means ± SEM. n = 4–12 animals.

Immunoblotting for phosphorylation status of insulin receptor (IR) or Akt showed that neither construct altered basal phosphorylation status of these proteins, nor the increase seen in response to the infusion (Fig. 5A and B). In the current study, there was also no difference in the basal phosphorylation state of ACC or the decrease in phosphorylation of ACC in response to hyperglycaemia in the SIRT1, or SIRT1 (H363Y), over expressing leg (Fig. 5C). This indicates that AMPK activity was unlikely to be affected by overexpression of SIRT1.

## Discussion

The current study provides evidence that increasing SIRT1 expression has no obvious impact on the development of glucose-induced insulin resistance in skeletal muscle. When SIRT1 or the deacetylase inactive version of SIRT1 (H363Y) were over expressed in muscle, there was no alteration of the insulin resistance that occurs with 5h of hyperglycaemia [12–14]. In previous *in vitro* studies, an increase in SIRT1 protein or activity was associated with a rescuing of the effects of incubation with high glucose (e.g. oxidative damage or wound healing [9,10]). One possibility for the differences between the previous *in vitro* and current *in vivo* studies is that *in vivo* activity of SIRT1 is tightly regulated by intracellular NAD^+^ content. Under such conditions it is possible that a decreased activity of SIRT1 cannot be overcome by simply overexpressing the protein. Other possibilities for the apparently different effects of SIRT1 overexpression are the origin of the cells used for *in vitro* studies (mesangial cells [9] or endothelial cells [8,10]), *in vivo* vs *in vitro* conditions, and/or duration of glucose exposure. Most studies, especially those in cells, use at least 24h of the hyperglycaemic insult, whereas our study examined 5h glucose infusion.

It has been reported previously that SIRT1 can modulate the insulin signaling pathway via repression of PTP1B [4], Rictor of the mTORC2 complex [27], or via regulation of the p85 [28], p55α and p50α [29] subunits of PI3K. In the current study we did not find any differences in the phosphorylation status of the insulin receptor, a target of PTP1B, or Akt, which is downstream of both mTORC2 and PI3K, either in the basal state or in response to hyperglycaemia. This indicates that one week of overexpression of SIRT1 protein is insufficient to modulate these indicators of insulin signaling pathway activity although this does not preclude that longer term increases in SIRT1 protein may have some effects.

Similarly, SIRT1 has been reported to regulate AMPK signaling through deacetylation and activation of its upstream kinase LKB1 [30]. Conversely, AMPK is thought to regulate SIRT1 activity by modulating NAD^+^ levels [31]. However phosphorylation of ACC, a well described target of AMPK, was not changed by overexpression of SIRT1 in either the basal state or in response to hyperglycaemia, suggesting unaltered AMPK signaling in this model. This lack of effect of SIRT1 on AMPK and ACC is consistent with other studies reporting no difference in the phosphorylation state or activity of AMPK and ACC when SIRT1 is specifically knocked out [29,32], or overexpressed [33] in muscle.

Consistent with previous *in vitro* studies associating SIRT1 with fatty acid oxidation [34–36], we found a small increase in palmitate oxidation using homogenates prepared from the SIRT1 overexpressing (test) leg. The mechanism mediating this increase remains unclear although mitochondrial content and metabolism in the basal state were unaffected by the increase in SIRT1 protein.

Although there is evidence for a role of SIRT1 in mitochondrial biogenesis, we show that there was also no change in markers of mitochondrial content, or function, in muscle after overexpression of SIRT1 for one week. Recently however, there has been some controversy about the role SIRT1 plays in mitochondrial biogenesis in muscle, especially *in vivo*. Supporting its role, a comprehensive study in C2C12 muscle cells showed that SIRT1, through its ability to deacetylate and activate PGC-1α, was intimately linked to mitochondrial biogenesis and function [35]. *In vivo*, studies in conditional whole body knockout [37], muscle specific knockout [38] as well as whole body over expression [37] of SIRT1 show similar links. Other studies by Philp *et al* [32] reported that muscle-specific knockout of SIRT1 does not alter the abundance or activities of complexes within the respiratory chain, or mitochondrial content. Interestingly though, when SIRT1 was overexpressed using a similar electroporation protocol to ours, there was a decrease in components of the respiratory chain [39,40] while a dominant-negative version of SIRT1 (H355Y) of SIRT1 increased these components [40]. More recently, transgenic overexpression of SIRT1 has reportedly no alteration in respiration rates of isolated mitochondria, with only small increases in some components of the respiratory chain (complex 2 and 4, cyt c) and no protection against the effects of a high fat diet [41]. Thus, the role of SIRT1 in regulating muscle mitochondrial function under normal conditions as well as in response to exercise or resveratrol treatment will remain controversial until the results of more studies using similar paradigms become available [42–44].

In a cultured cell system we showed that overexpression of our SIRT1 construct led to a reduced acetylation of p53 using an antibody directed against acetylated p53 (K379). This demonstrated that the construct produced a functional SIRT1 enzyme capable of altering protein acetylation. In control and SIRT1 overexpressing TC muscle the same antibody did not detect any p53 acetylation and therefore there is no direct evidence that the SIRT1 protein produced in TC muscle was capable of altering protein acetylation. However, we have used the electroporation protocol previously to overexpress many functional proteins [17–19] and see no reason to suspect that the SIRT1 enzyme would not be active in TC muscle if substrate availability and other potential regulatory factors were favourable. It is possible that tight regulation of SIRT1 activity via substrate (NAD^+^) availability occurs *in vivo*, explaining the lack of effect of simply increasing the amount of SIRT1 protein.

In conclusion, our results suggest that although a decrease in SIRT1 may be involved in the development of insulin resistance in skeletal muscle in response to hyperglycaemia, overexpressing SIRT1 does not alleviate this insulin resistance. This data also shows that increasing SIRT1 protein has little effect on muscle mitochondrial biogenesis *in vivo*. The acetylation/sirtuin axis is clearly complex and changes in SIRT1 protein may not equate to linear changes in enzyme activity, due to requirement for NAD^+^ as a co-factor. Therefore strategies aimed at raising NAD^+^ levels [45–47] might be more useful in examining the role of SIRT1 in the acute regulation of muscle metabolism in response to a hyperglycaemic insult.

## References

[1] Nogueiras R, Habegger KM, Chaudhary N, Finan B, Banks AS, Dietrich MO, et al Sirtuin 1 and sirtuin 3: physiological modulators of metabolism. Physiol Rev 2012;92: 1479–1514. 10.1152/physrev.00022.2011 22811431 PMC3746174

[2] Bordone L, Cohen D, Robinson A, Motta MC, van Veen E, Czopik A, et al SIRT1 transgenic mice show phenotypes resembling calorie restriction. Aging Cell 2007;6: 759–767. 17877786 10.1111/j.1474-9726.2007.00335.x

[3] Boily G, Seifert EL, Bevilacqua L, He XH, Sabourin G, Estey C, et al SirT1 regulates energy metabolism and response to caloric restriction in mice. PLoS One 2008;3: e1759 10.1371/journal.pone.0001759 18335035 PMC2258149

[4] Sun C, Zhang F, Ge X, Yan T, Chen X, Shi X, et al SIRT1 improves insulin sensitivity under insulin-resistant conditions by repressing PTP1B. Cell Metab 2007;6: 307–319. 17908559 10.1016/j.cmet.2007.08.014

[5] Rodgers JT, Lerin C, Haas W, Gygi SP, Spiegelman BM, Puigserver P. Nutrient control of glucose homeostasis through a complex of PGC-1alpha and SIRT1. Nature 2005;434: 113–118. 15744310 10.1038/nature03354

[6] Suchankova G, Nelson LE, Gerhart-Hines Z, Kelly M, Gauthier MS, Saha AK, et al Concurrent regulation of AMP-activated protein kinase and SIRT1 in mammalian cells. Biochem Biophys Res Commun 2009;378: 836–841. 10.1016/j.bbrc.2008.11.130 19071085 PMC2764524

[7] Balestrieri ML, Rienzo M, Felice F, Rossiello R, Grimaldi V, Milone L, et al High glucose downregulates endothelial progenitor cell number via SIRT1. Biochim Biophys Acta 2008;1784: 936–945. 10.1016/j.bbapap.2008.03.004 18423418

[8] Yuan Q, Chen L, Xiang DX, Li YJ, Hu CP. Effect of resveratrol derivative BTM-0512 on high glucose-induced dysfunction of endothelial cells: role of SIRT1. Can J Physiol Pharmacol 2011;89: 713–722. 10.1139/y11-069 21905824

[9] Xu Y, Nie L, Yin YG, Tang JL, Zhou JY, Li DD, et al Resveratrol protects against hyperglycemia-induced oxidative damage to mitochondria by activating SIRT1 in rat mesangial cells. Toxicol Appl Pharmacol 2012;259: 395–401. 10.1016/j.taap.2011.09.028 22015446

[10] Wang Y, Zhao X, Shi D, Chen P, Yu Y, Yang L, et al Overexpression of SIRT1 Promotes High Glucose-Attenuated Corneal Epithelial Wound Healing via p53 Regulation of the IGFBP3/IGF-1R/AKT Pathway. Invest Ophthalmol Vis Sci 2013;54: 3806–3814. 10.1167/iovs.13-12091 23661372

[11] Nedachi T, Kadotani A, Ariga M, Katagiri H, Kanzaki M. Ambient glucose levels qualify the potency of insulin myogenic actions by regulating SIRT1 and FoxO3a in C2C12 myocytes. Am J Physiol Endocrinol Metab 2008;294: E668–678. 10.1152/ajpendo.00640.2007 18230695

[12] Brandon AE, Hoy AJ, Wright LE, Turner N, Hegarty BD, Iseli TJ, et al The evolution of insulin resistance in muscle of the glucose infused rat. Arch Biochem Biophys 2011;509: 133–141. 10.1016/j.abb.2011.03.008 21420928 PMC3087290

[13] Hoy AJ, Bruce CR, Cederberg A, Turner N, James DE, Cooney GJ, et al Glucose infusion causes insulin resistance in skeletal muscle of rats without changes in Akt and AS160 phosphorylation. Am J Physiol Endocrinol Metab 2007;293: E1358–1364. 17785505 10.1152/ajpendo.00133.2007

[14] Kraegen EW, Saha AK, Preston E, Wilks D, Hoy AJ, Cooney GJ, et al Increased malonyl-CoA and diacylglycerol content and reduced AMPK activity accompany insulin resistance induced by glucose infusion in muscle and liver of rats. American Journal of Physiology—Endocrinology & Metabolism 2006;290: E471–479. 16234268 10.1152/ajpendo.00316.2005

[15] Saha AK, Xu XJ, Lawson E, Deoliveira R, Brandon AE, Kraegen EW, et al Downregulation of AMPK accompanies leucine- and glucose-induced increases in protein synthesis and insulin resistance in rat skeletal muscle. Diabetes 2010;59: 2426–2434. 10.2337/db09-1870 20682696 PMC3279521

[16] Zu Y, Liu L, Lee MY, Xu C, Liang Y, Man RY, et al SIRT1 promotes proliferation and prevents senescence through targeting LKB1 in primary porcine aortic endothelial cells. Circ Res 2010;106: 1384–1393. 10.1161/CIRCRESAHA.109.215483 20203304

[17] Bruce CR, Brolin C, Turner N, Cleasby ME, van der Leij FR, Cooney GJ, et al Overexpression of carnitine palmitoyltransferase I in skeletal muscle in vivo increases fatty acid oxidation and reduces triacylglycerol esterification. American Journal of Physiology—Endocrinology & Metabolism 2007;292: E1231–1237. 17179390 10.1152/ajpendo.00561.2006

[18] Cleasby ME, Davey JR, Reinten TA, Graham MW, James DE, Kraegen EW, et al Acute bidirectional manipulation of muscle glucose uptake by in vivo electrotransfer of constructs targeting glucose transporter genes. Diabetes 2005;54: 2702–2711. 16123360 10.2337/diabetes.54.9.2702

[19] Wright LE, Brandon AE, Hoy AJ, Forsberg GB, Lelliott CJ, Reznick J, et al Amelioration of lipid-induced insulin resistance in rat skeletal muscle by overexpression of Pgc-1beta involves reductions in long-chain acyl-CoA levels and oxidative stress. Diabetologia 2011. 10.1007/s00125-011-2068-x 21331471

[20] McMahon JM, Signori E, Wells KE, Fazio VM, Wells DJ. Optimisation of electrotransfer of plasmid into skeletal muscle by pretreatment with hyaluronidase—increased expression with reduced muscle damage. Gene Ther 2001;8: 1264–1270. 11509960 10.1038/sj.gt.3301522

[21] Hoy AJ, Brandon AE, Turner N, Watt MJ, Bruce CR, Cooney GJ, et al Lipid and insulin infusion-induced skeletal muscle insulin resistance is likely due to metabolic feedback and not changes in IRS-1, Akt or AS160 phosphorylation. Am J Physiol Endocrinol Metab 2009: E67–75. 10.1152/ajpendo.90945.2008 19366875 PMC2711668

[22] James DE, Jenkins AB, Kraegen EW. Heterogeneity of insulin action in individual muscles in vivo: euglycemic clamp studies in rats. American Journal of Physiology 1985;248: E567–574. 3887942 10.1152/ajpendo.1985.248.5.E567

[23] Turner N, Bruce CR, Beale SM, Hoehn KL, So T, Rolph MS, et al Excess lipid availability increases mitochondrial fatty acid oxidative capacity in muscle: evidence against a role for reduced fatty acid oxidation in lipid-induced insulin resistance in rodents. Diabetes 2007;56: 2085–2092. 17519422 10.2337/db07-0093

[24] Montgomery MK, Osborne B, Brown SH, Small L, Mitchell TW, Cooney GJ, et al Contrasting metabolic effects of medium- versus long-chain fatty acids in skeletal muscle. J Lipid Res 2013;54: 3322–3333. 10.1194/jlr.M040451 24078708 PMC3826680

[25] Larsen S, Stride N, Hey-Mogensen M, Hansen CN, Bang LE, Bundgaard H, et al Simvastatin effects on skeletal muscle: relation to decreased mitochondrial function and glucose intolerance. J Am Coll Cardiol 2013;61: 44–53. 10.1016/j.jacc.2012.09.036 23287371

[26] Shabalina IG, Petrovic N, de Jong JM, Kalinovich AV, Cannon B, Nedergaard J. UCP1 in brite/beige adipose tissue mitochondria is functionally thermogenic. Cell Rep 2013;5: 1196–1203. 10.1016/j.celrep.2013.10.044 24290753

[27] Wang RH, Kim HS, Xiao C, Xu X, Gavrilova O, Deng CX. Hepatic Sirt1 deficiency in mice impairs mTorc2/Akt signaling and results in hyperglycemia, oxidative damage, and insulin resistance. J Clin Invest 2011;121: 4477–4490. 10.1172/JCI46243 21965330 PMC3204833

[28] Frojdo S, Durand C, Molin L, Carey AL, El-Osta A, Kingwell BA, et al Phosphoinositide 3-kinase as a novel functional target for the regulation of the insulin signaling pathway by SIRT1. Mol Cell Endocrinol 2011;335: 166–176. 10.1016/j.mce.2011.01.008 21241768

[29] Schenk S, McCurdy CE, Philp A, Chen MZ, Holliday MJ, Bandyopadhyay GK, et al Sirt1 enhances skeletal muscle insulin sensitivity in mice during caloric restriction. J Clin Invest 2011;121: 4281–4288. 10.1172/JCI58554 21985785 PMC3204844

[30] Lan F, Cacicedo JM, Ruderman N, Ido Y. SIRT1 modulation of the acetylation status, cytosolic localization, and activity of LKB1. Possible role in AMP-activated protein kinase activation. J Biol Chem 2008;283: 27628–27635. 10.1074/jbc.M805711200 18687677 PMC2562073

[31] Canto C, Auwerx J. PGC-1alpha, SIRT1 and AMPK, an energy sensing network that controls energy expenditure. Curr Opin Lipidol 2009;20: 98–105. 10.1097/MOL.0b013e328328d0a4 19276888 PMC3627054

[32] Philp A, Chen A, Lan D, Meyer GA, Murphy AN, Knapp AE, et al Sirtuin 1 (SIRT1) deacetylase activity is not required for mitochondrial biogenesis or peroxisome proliferator-activated receptor-gamma coactivator-1alpha (PGC-1alpha) deacetylation following endurance exercise. J Biol Chem 2011;286: 30561–30570. 10.1074/jbc.M111.261685 21757760 PMC3162416

[33] White AT, McCurdy CE, Philp A, Hamilton DL, Johnson CD, Schenk S. Skeletal muscle-specific overexpression of SIRT1 does not enhance whole-body energy expenditure or insulin sensitivity in young mice. Diabetologia 2013;56: 1629–1637. 10.1007/s00125-013-2912-2 23604553 PMC3703320

[34] Feige JN, Lagouge M, Canto C, Strehle A, Houten SM, Milne JC, et al Specific SIRT1 activation mimics low energy levels and protects against diet-induced metabolic disorders by enhancing fat oxidation. Cell Metab 2008;8: 347–358. 10.1016/j.cmet.2008.08.017 19046567

[35] Gerhart-Hines Z, Rodgers JT, Bare O, Lerin C, Kim SH, Mostoslavsky R, et al Metabolic control of muscle mitochondrial function and fatty acid oxidation through SIRT1/PGC-1alpha. Embo J 2007;26: 1913–1923. 17347648 10.1038/sj.emboj.7601633 PMC1847661

[36] Purushotham A, Schug TT, Xu Q, Surapureddi S, Guo X, Li X. Hepatocyte-specific deletion of SIRT1 alters fatty acid metabolism and results in hepatic steatosis and inflammation. Cell Metab 2009;9: 327–338. 10.1016/j.cmet.2009.02.006 19356714 PMC2668535

[37] Price NL, Gomes AP, Ling AJ, Duarte FV, Martin-Montalvo A, North BJ, et al SIRT1 is required for AMPK activation and the beneficial effects of resveratrol on mitochondrial function. Cell Metab 2012;15: 675–690. 10.1016/j.cmet.2012.04.003 22560220 PMC3545644

[38] Menzies KJ, Singh K, Saleem A, Hood DA. Sirtuin 1-mediated effects of exercise and resveratrol on mitochondrial biogenesis. J Biol Chem 2013;288: 6968–6979. 10.1074/jbc.M112.431155 23329826 PMC3591607

[39] Gurd BJ, Yoshida Y, Lally J, Holloway GP, Bonen A. The deacetylase enzyme SIRT1 is not associated with oxidative capacity in rat heart and skeletal muscle and its overexpression reduces mitochondrial biogenesis. J Physiol 2009;587: 1817–1828. 10.1113/jphysiol.2008.168096 19237425 PMC2683967

[40] Higashida K, Kim SH, Jung SR, Asaka M, Holloszy JO, Han DH. Effects of Resveratrol and SIRT1 on PGC-1alpha Activity and Mitochondrial Biogenesis: A Reevaluation. PLoS Biol 2013;11: e1001603 10.1371/journal.pbio.1001603 23874150 PMC3706311

[41] White AT, Philp A, Fridolfsson HN, Schilling JM, Murphy AN, Hamilton DL, et al High-fat diet-induced impairment of skeletal muscle insulin sensitivity is not prevented by SIRT1 overexpression. Am J Physiol Endocrinol Metab 2014;307: E764–E772. 10.1152/ajpendo.00001.2014 25159328 PMC4216952

[42] Menzies KJ, Chabi B, Hood DA, Schenk S, Philp A, Braga VA, et al Commentaries on viewpoint: does SIRT1 determine exercise-induced skeletal muscle mitochondrial biogenesis: differences between in vitro and in vivo experiments? J Appl Physiol (1985) 2012;112: 929–930. 10.1152/japplphysiol.00094.2012 22383496

[43] Gurd BJ, Little JP, Perry CG. Does SIRT1 determine exercise-induced skeletal muscle mitochondrial biogenesis: differences between in vitro and in vivo experiments? J Appl Physiol (1985) 2012;112: 926–928. 10.1152/japplphysiol.01262.2011 22096123

[44] Gurd BJ, Little JP, Perry CG. Last word on viewpoint: does SIRT1 determine exercise-induced skeletal muscle mitochondrial biogenesis: differences between in vitro and in vivo experiments? J Appl Physiol (1985) 2012;112: 931 10.1152/japplphysiol.00078.2012 22383497

[45] Canto C, Houtkooper RH, Pirinen E, Youn DY, Oosterveer MH, Cen Y, et al The NAD(+) precursor nicotinamide riboside enhances oxidative metabolism and protects against high-fat diet-induced obesity. Cell Metab 2012;15: 838–847. 10.1016/j.cmet.2012.04.022 22682224 PMC3616313

[46] Bai P, Canto C, Brunyanszki A, Huber A, Szanto M, Cen Y, et al PARP-2 regulates SIRT1 expression and whole-body energy expenditure. Cell Metab 2011;13: 450–460. 10.1016/j.cmet.2011.03.013 21459329 PMC3108571

[47] Bai P, Canto C, Oudart H, Brunyanszki A, Cen Y, Thomas C, et al PARP-1 inhibition increases mitochondrial metabolism through SIRT1 activation. Cell Metab 2011;13: 461–468. 10.1016/j.cmet.2011.03.004 21459330 PMC3086520

